# Congenital Coronary Artery Anomalies: Three Cases and Brief Review of the Literature

**DOI:** 10.1155/2021/6612289

**Published:** 2021-01-30

**Authors:** Nikolaos S. Ioakeimidis, Dimitrios Valasiadis, Andreas Markou, Theodora Zaglavara

**Affiliations:** ^1^Department of Cardiology, General Hospital of Florina “Eleni Th. Dimitriou”, Egnatias 9, Florina 53100, Greece; ^2^Department of Radiology, General Hospital of Florina “Eleni Th. Dimitriou”, Egnatias 9, Florina 53100, Greece; ^3^Department of Cardiovascular Imaging, Interbalkan Medical Centre of Thessaloniki, Asklipiou 10, Thessaloniki 57001, Greece

## Abstract

Coronary artery anomalies (CAAs) are congenital vascular defects which can remain hidden and asymptomatic over the complete life course of an individual. They are defined as deviations from the normal coronary anatomy regarding the arterial origin, course, or both. Their incidence varies from 1.3% to 5.64% in coronary angiography cohorts, and they can be detected as incidental findings. In certain cases, CAAs can be hemodynamically significant and unfortunately can be proven lethal. Their link with sudden cardiac death, especially in otherwise healthy competitive athletes, is well established, but their prognostic significance, range of symptoms, and pathophysiology remain to be further elucidated. Here, along with a brief review of related literature, we present a series of three cases: one case of an anomalous origin of the right coronary artery (RCA) from the left coronary sinus, one case of a split RCA originating from the left coronary sinus, and one case of a dual left anterior descending (LAD) artery system.

## 1. Introduction and Brief Review of the Literature

Coronary artery anomalies (CAAs) are congenital defects whose hemodynamic significance, clinical presentation, and prognosis remain challenging to define. Their diagnosis is mostly incidental, and the range of symptoms they can produce is broad, varying from dyspnea to anginal complaints [1]. Numerous reports regarding their hemodynamic significance and possible lethality emerged in the 1970s, and since then, there is a growing interest to further elucidate their pathophysiology [2, 3]. Most importantly, CAAs have drawn the attention of the medical community due to the association of specific anatomical subtypes with sudden cardiac death (SCD), especially in young competitive and otherwise “healthy” athletes [4–6]. A CAA is characterized as such by taking into consideration the expected normal coronary anatomy and any possible abnormality regarding coronary artery origin, course, or both [1]. CAAs affect almost 1% of the general population, and their prevalence is found to be lower in necropsies [7]. In a cohort of 1950 patients who underwent coronary angiography, strict anatomical criteria were applied and the incidence of CAAs was found to be 5.64% [8]. Another retrospective study of 2572 multidetector computed tomography (MDCT) coronary angiograms revealed a prevalence of 2.33% [9]. The observed discrepancies between prevalence and incidence values are probably attributed to differences in the diagnostic definitions of CAAs, with some being more lenient and others stricter. Angelini et al., after careful scrutiny, provide a detailed classification of CAAs, which serves as a basis for further investigation of their clinical significance, and identify broad categories: anomalies of origination and course, anomalies of intrinsic coronary arterial anatomy, anomalies of coronary termination, and anomalous collateral vessels [7]. In their view, the characterization of anomalies as “minor” or “major,” based on their clinical consequences, seems futile since large prospective studies regarding the prognostic impact of distinct CAAs are lacking. Yamanaka and Hobbs report an incidence of 1.3% in a cohort of 126,595 patients who underwent coronary angiography [10]. In their study, 87% of patients with a CAA had anomalous origin and distribution and the remaining 13% had coronary artery fistulae. The most common CAAs provided in order of decreasing incidence are split right coronary artery (RCA), ectopic RCA from the right coronary cusp, ectopic RCA from the left cusp, coronary fistulas, absent left main coronary artery (LMCA), circumflex coronary artery from the right cusp, left coronary artery (LCA) arising from the right cusp, and low origination of the RCA [7]. Regarding the link of CAAs with sudden cardiac death, it has been found that 0.6% of sudden death cases in apparently healthy young individuals could be attributed to a CAA [11]. The most common malignant anomalies reported were LCA arising from the right coronary sinus (which correlates with SCD during exertion) and RCA arising from the left coronary sinus, both following an interarterial course between the aorta and the pulmonary artery [11]. CAAs are reported as a cause of SCD in a series of young competitive athletes, being the third most common cause after definite hypertrophic cardiomyopathy (HCM) and probable HCM, accounting for 13% of deaths [12]. In the aforementioned series, the most common malignant anomaly was a left main coronary artery arising from the right coronary sinus with an interarterial course. Altered flow patterns within the anomalous vessel, which can lead to thrombus formation, reduced coronary reserve, and strangulation of the anomalous vessel by neighboring structures under specific circumstances (as in the case of an anomalous LCA with an interarterial course), have all been implicated in the pathophysiology of CAAs, trying to explain their spectrum of symptoms [7].

Here, we present a total of three cases, more specifically one case with an anomalous origin of the RCA from the left coronary sinus, one case with a split RCA originating from the left coronary sinus, and one case of a dual left anterior descending (LAD) artery system.

## 2. Case Presentations

### 2.1. Case 1: Anomalous Origin of the RCA from the Left Coronary Sinus

**Figure 1:**
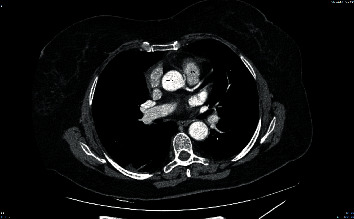
CT angiography of the aorta revealing an anomalous origin of the RCA (depicted with an “∗**”**). The RCA exhibits an interarterial course between the aorta and the pulmonary artery (PA).

**Figure 2:**
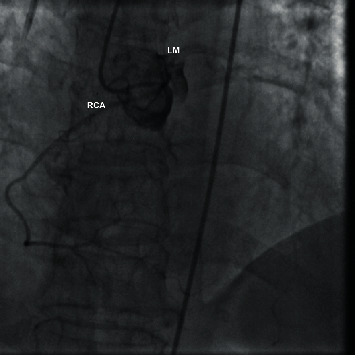
Coronary angiography confirming the anomalous aortic origin of the RCA from the left coronary sinus. LM: left main.

**Figure 3:**
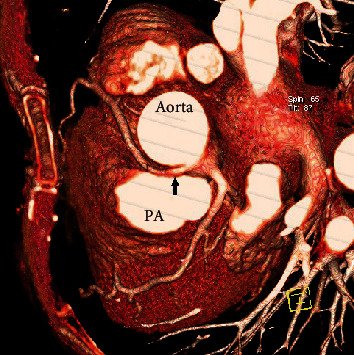
CT coronary angiography with volume-rendered 3D reconstruction of the images, revealing the interarterial course of the RCA (pointed with the arrow) between the aorta and the pulmonary artery (PA).

We present the case of a 70-year-old woman who presented at the emergency department (ED) of our non-percutaneous coronary intervention- (non-PCI-) capable hospital with a complaint of exertional dyspnea and a stabbing retrosternal pain radiating between her shoulders, both bothering her intermittently over the past three hours. The patient was overweight, and her medical history included arterial hypertension and hypothyroidism both controlled with medication. The 12-lead ECG revealed a normal sinus rhythm with ST segment depression of 1 mm at the anterior and lateral leads. High sensitivity troponin-I (hs-cTnI) was measured before admission to the coronary care unit (CCU) at 83.2 ng/L (normal value < 29 ng/L), confirming the diagnosis of non-ST elevation myocardial infarction (NSTEMI). The rest of the blood and biochemical panel was within normal limits, including a negative D-dimer assay. Chest X-ray revealed a marginally increased cardiothoracic ratio, and transthoracic echocardiography (TTE) revealed mild concentric left ventricular (LV) wall hypertrophy with a normal left ventricular ejection fraction (LVEF) and no wall motion abnormalities. Before the initiation of antiplatelets and due to the clinical description of the patient's complaint, a computed tomography (CT) angiography of the aorta was performed in order to rule out an acute aortic syndrome. No signs of aortic pathology were detected, but the CT revealed an anomalous origin of the RCA from the left coronary sinus (Figure 1). The ectopic RCA followed an interarterial course between the trunk of the pulmonary artery and the aorta. It should be noted that the CT scanner used was not technically capable of performing a CT coronary angiography (CTCA) but adequately visualized the anomaly. The patient remained hemodynamically stable during her stay, her symptoms gradually improved, and hs-cTnI was normalized. The patient was referred to a tertiary hospital for coronary angiography which confirmed the anomalous origin of the RCA (Figure 2) and did not reveal any atherosclerotic coronary lesions. A CTCA was also performed, after the classical coronary angiography, to accurately visualize the anatomy of the CAA (Figure 3). The CTCA unveiled an eccentric noncalcified plaque at the middle segment of the LAD branch of the LCA, causing a luminal stenosis of 25-49%, which we speculate is the culprit lesion of the patient's NSTEMI, probably involving a mechanism of plaque rupture and subsequent fibrinolysis or coronary artery spasm. Finally, a dobutamine stress echocardiography study was negative for segmental wall motion abnormalities. In this case, the CAA was an incidental finding on the initial CT aortic angiography. It is probably a benign finding since the observed ischemic changes of the ECG were not compatible with the supply distribution of the RCA, and the stress echo study was unremarkable.

### 2.2. Case 2: Split RCA with an Anomalous Origin from the Left Coronary Sinus

**Figure 4:**
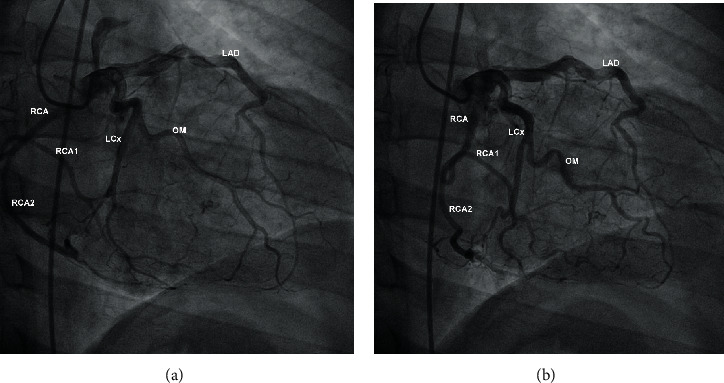
(a, b) Coronary angiography (right anterior oblique projection) revealing the split RCA (marked as RCA1 and RCA2) with an anomalous origin from the left coronary sinus. The RCA and LCA were outlined simultaneously using the JL4 catheter placed at the left coronary sinus. LCx: left circumflex artery; OM: obtuse marginal artery; LAD: left anterior descending (artery).

We present the case of a 44-year-old man who presented at the ED of our non-PCI-capable hospital with a complaint of an intermittent burning-like sensation at the precordium during physical activity over the past two days. The patient was overweight and reported a smoking history of 30 pack-years. He was not taking any medication, and the rest of his medical history was unremarkable. The ECG revealed a normal sinus rhythm with anterior, high-lateral, and lateral repolarization disturbances (shallow inverted T waves at leads I, aVL, and V3 to V6). A measurement of hs-cTnI yielded a value of 38 ng/L (normal value < 29 ng/L), whereas the rest of the complete blood count and biochemical panel was unremarkable, apart from dyslipidemia (total cholesterol = 252 mg/dL, LDL = 155 mg/dL, and TG = 262 mg/dL). TTE revealed a reduced LVEF of 40%, increased end-diastolic LV diameter, and hypokinesis of the LV anterior, lateral, and inferolateral walls. The diagnosis of NSTEMI was confirmed; the patient was admitted to the CCU and initiated on dual antiplatelet therapy (DAPT) with aspirin and clopidogrel, low molecular weight heparin, b-blocker, ACE inhibitor, and a statin. His stay was uneventful with gradual remission of his complaint and improvement of his LV wall motion abnormalities. He was referred to a tertiary hospital for coronary angiography which revealed no atherosclerotic lesions and a split RCA with an anomalous origin from the left coronary sinus (Figures 4(a) and 4(b)). The RCA featured an initial common trunk, which then split into two distinct segments of similar caliber with one of them (RCA1 in Figures 4(a) and 4(b)) probably following a posterior course. The definition of a split RCA or a double RCA is a matter of great controversy with various anatomical descriptions, and no consensus has been reached so far [13, 14]. Unfortunately, after thorough discussion, the patient was not willing to undergo a CTCA which would visualize the anomaly in greater detail. Since coronary angiography did not reveal any atherosclerotic lesions, the patient's acute coronary syndrome (ACS) could be characterized as a myocardial infarction with nonobstructive coronary arteries (MINOCA), and based on existing evidence [15], DAPT with aspirin and clopidogrel was continued (aiming for a maximum of one year) in conjunction with an ACE inhibitor, a b-blocker, and a statin. Upon follow-up echocardiography, three months after angiography, an evident improvement of LVEF was observed (55%) along with restoration of normal wall motion.

### 2.3. Case 3: A Dual LAD System

**Figure 5:**
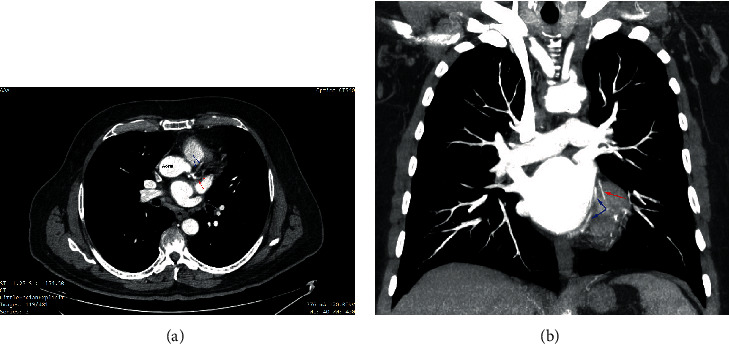
CT angiography of the aorta revealing a short LMCA (a) which then splits into two distinct branches (pointed with arrows of different color) descending toward the apex in the anterior interventricular groove. The short LAD is pointed with blue arrows and the long LAD with red arrows.

**Figure 6:**
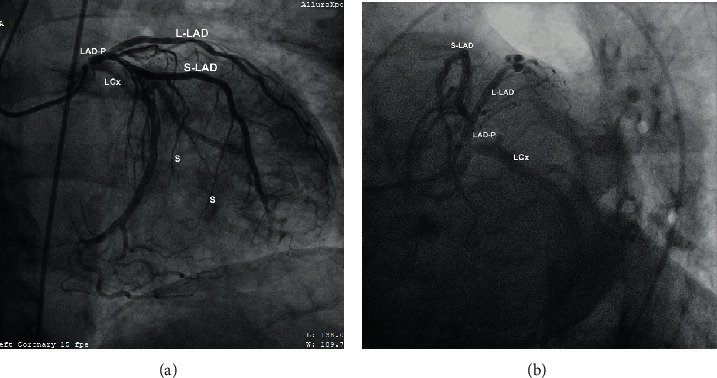
(a, b) Coronary angiogram: right anterior oblique caudal projection (a) and left anterior oblique caudal “spider” projection (b). A very short LMCA, best visualized in (b), gives rise to the LCx and a common LAD trunk which we denote as LAD-P. The LAD-P bifurcates into two arteries, S-LAD, giving rise to long septal perforators, and L-LAD reaching the apex, giving rise to shorter perforating arteries and diagonals. LAD-P: left anterior descending proper; L-LAD: long LAD; S-LAD: short LAD; LCx: left circumflex artery; S: septal perforating branches.

We present the case of a 60-year-old man presenting at the ED of our non-PCI-capable hospital with a complaint of breathlessness at rest and on exertion over the past two days. The patient's medical history was unremarkable apart from obesity. Upon presentation, he was hemodynamically stable, and clinical examination revealed moderate wheezing and diffuse rales at lung bases bilaterally on auscultation (Killip Class II). His ECG revealed a normal sinus rhythm with a left anterior fascicular block (LAFB); shallow T wave inversion at leads I, aVL, and V6; and T wave flattening at lead V5. A measurement of cardiac troponin T (cTnT) yielded a value of 71 ng/L (normal value < 40 ng/L), and the diagnosis of a NSTEMI was highly likely. TTE revealed a reduced LVEF of 40% with no regional wall motion abnormalities. However, a difference of blood pressure values of about 20 mmHg between the two arms prompted a CT angiography of the aorta before the initiation of antiplatelet therapy. It revealed no aortic pathology but visualized a short LMCA which then split into two distinct branches of the same caliber descending toward the apex in the anterior interventricular groove, compatible with the course of the LAD artery (Figure 5). The patient was started on antiplatelets and intravenous diuretics. His stay at the CCU remained uneventful with gradual remission of symptoms and improvement of myocardial enzymes. He was referred to a tertiary center for coronary angiography which revealed an atherosclerotic lesion at the middle segment of the LCx causing a luminal stenosis of about 40% and one lesion at the second obtuse marginal branch of the LCx also causing a stenosis of 40%. Moreover, a short LMCA was confirmed (Figure 6(b), left anterior oblique caudal “spider” view), which in turn gave rise to the LCx and a common LAD trunk (which we denote as LAD-proper). The proper LAD bifurcated into two arteries, one giving rise to long septal perforators (short LAD) and one reaching the apex (long LAD), giving rise to shorter perforating arteries and diagonals (Figures 6(a) and 6(b)). The patient was discharged on DAPT (aiming for a maximum duration of one year), an ACE inhibitor, a b-blocker, a statin, and furosemide. Follow-up echocardiography was performed three months post angiography revealing a markedly improved LVEF of 52%.

Spindola-Franco et al., in 1983, classified dual LAD variants into distinct types (Types I, II, III, and IV) by studying a cohort of 2140 patients who underwent coronary angiography [16]. The prevalence of a dual LAD was 1% in their study (23 patients). Our case fits the angiographic description of a Type I dual LAD according to the abovementioned classification. Since the original classification of Spindola-Franco et al., three more dual LAD types have been described in the literature, raising the total types to seven [17, 18]. Regarding our current case, the opinion of expert interventional cardiologists who were asked to evaluate the angiographic findings was wavering between a dual LAD and a long diagonal branch, a situation that was to be expected considering the rarity of such a variant.

## 3. Discussion

Coronary artery anomalies remain an established cause of SCD. Presymptomatic screening protocols have been proposed especially for high-risk individuals (e.g., competitive athletes) by using novel imaging modalities like cardiac magnetic resonance [1]. The accumulation of data and the prospective study of large cohorts with CAAs, comprised of individuals detected through screening, could further elucidate the prognostic impact and pathophysiology of CAAs. The identification of highly malignant variants could prompt an early surgical intervention.

Moreover, CAAs should be taken into account in cases of an acute coronary syndrome or within the context of anginal symptoms. Considering the fact that all three patients presented to a non-PCI- and non-CTCA-capable hospital, the anomalies of Case 1 and Case 3 were discovered incidentally on CT, which was prompted by emergent clinical suspicion, thus not delaying invasive management. In our view and according to the published literature, CAAs can be divided into hemodynamically significant and nonhemodynamically significant [19]. Duplication of the RCA or LAD, like in Cases 2 and 3, respectively, are considered nonhemodynamically significant anomalies, but they may perplexing surgical interventions such as coronary artery bypass grafting or percutaneous coronary interventions especially if both vessels are diseased. An interarterial course, like in Case 1 of our series, is considered significant since it can produce a wide range of symptoms under specific load circumstances. Case 1 could be classified as a MINOCA, and although the observed ischemic changes of the ECG were not compatible with the supply distribution of the ectopic RCA, we sought to investigate the “behavior” of the anomaly under increased load. This is one of the reasons we performed a stress-echo study which proved to be normal. Regarding Case 1 of our series, one could argue that a cardiac magnetic resonance (CMR) study was the imaging modality of choice to evaluate any eventual myocardial inflammation or fibrosis and simultaneously visualize the coronary anomaly. The reason it was not performed was purely financial from the patient's perspective. For the same case, an intravascular imaging modality, like optical coherence tomography (OCT) or intravascular ultrasound (IVUS), could have been used in conjunction with intracoronary functional testing in order to elucidate the behavior of the mid-LAD stenosis (detected on CTCA and graded as 25-49%) and also confirm the diagnosis of MINOCA with greater confidence. However, considering the ALARA (as low as reasonably achievable) principle of radioprotection, the patient had already undergone a CT aortography at the time of initial presentation (using a non-CTCA-capable scanner), then a coronary angiography which revealed no stenosis (therefore, no intracoronary imaging or functional tests were performed), and finally a CTCA. Subsequently, a second coronary angiography with OCT or IVUS and intracoronary functional testing was deemed excessive since the patient was stable and asymptomatic and was receiving optimal medical therapy upon follow-up examination. Regarding Case 2 (no angiographic stenosis) and Case 3 (moderate coronary atherosclerotic lesions), a thorough assessment with multimodality imaging (CMR, IVUS or OCT, and coronary functional testing) could safely confirm or exclude the diagnosis of MINOCA, according to published scientific statements [20]. However, multimodality imaging is not readily available at every tertiary site in the Greek region, which was the case for these patients. It should also be noted that the choice of a femoral catheterization approach for all three cases was based on operator experience and procedural confidence.

## Data Availability

Source data used to support the findings of this study, more specifically imaging studies and laboratory investigation results, are restricted by Greek (Law 4624/2019) and European [(EU) 2016/679] regulation in order to protect patient health data as well as their privacy and anonymity. Data are available upon precisely reasoned application to the Scientific Council of the General Hospital of Florina “Eleni Th. Dimitriou,” which is a public hospital of the Greek National Health System and can be contacted at postal address Egnatias 9, Florina, 53100, Greece, for researchers who meet the criteria to access confidential data.
